# The lemur tail kinase family in neuronal function and disfunction in neurodegenerative diseases

**DOI:** 10.1007/s00018-024-05480-0

**Published:** 2024-11-09

**Authors:** Angelique Larose, Christopher C. J. Miller, Gábor M. Mórotz

**Affiliations:** 1 Department of Pharmacology and Pharmacotherapy, Semmelweis University, Nagyvárad tér 4, Budapest, H-1089 Hungary; 2 Center for Pharmacology and Drug Research & Development, Semmelweis University, Budapest, Hungary; 3 Department of Basic and Clinical Neuroscience, Institute of Psychiatry, Psychology and Neuroscience, King’s College London, 125 Coldharbour Lane Camberwell, London, SE5 9RX UK

**Keywords:** Lemur tail kinase, Cyclin dependent kinase-5/p35, Glycogen synthase kinase-3 beta, Axonal transport, Endosome, Alzheimer’s disease

## Abstract

The complex neuronal architecture and the long distance of synapses from the cell body require precisely orchestrated axonal and dendritic transport processes to support key neuronal functions including synaptic signalling, learning and memory formation. Protein phosphorylation is a major regulator of both intracellular transport and synaptic functions. Some kinases and phosphatases such as cyclin dependent kinase-5 (cdk5)/p35, glycogen synthase kinase-3β (GSK3β) and protein phosphatase-1 (PP1) are strongly involved in these processes. A primary pathological hallmark of neurodegenerative diseases, including Alzheimer’s disease, Parkinson’s disease and amyotrophic lateral sclerosis/frontotemporal dementia, is synaptic degeneration together with disrupted intracellular transport. One attractive possibility is that alterations to key kinases and phosphatases may underlie both synaptic and axonal transport damages. The brain enriched lemur tail kinases (LMTKs, formerly known as lemur tyrosine kinases) are involved in intracellular transport and synaptic functions, and are also centrally placed in cdk5/p35, GSK3β and PP1 signalling pathways. Loss of LMTKs is documented in major neurodegenerative diseases and thus can contribute to pathological defects in these disorders. However, whilst function of their signalling partners became clearer in modulating both synaptic signalling and axonal transport progress has only recently been made around LMTKs. In this review, we describe this progress with a special focus on intracellular transport, synaptic functions and neurodegenerative diseases.

## Introduction

Neurons represent some of the most complex and architecturally elaborate cells of eukaryotes. Specialised synaptic connections that mediate neuron to neuron signalling serve to compute and transmit information to effector cells and tissues, and the polarised structure of neurons with axons and dendrites places unique demands on intracellular transport processes. Most neuronal proteins are synthesised in cell bodies and so decisions have to be made as to whether cargoes are transported to axons, dendrites or both compartments. Even within axons cargoes need to be delivered to different destinations; for example synaptic vesicle precursors are delivered to presynaptic terminals whereas Na^+^ channels are enriched in nodes of Ranvier. Finally, as neurons represent some of the largest cells, this transport can involve especially long distances; human motor neuron can be over 1 m in length. It is generally accepted that like synaptic function the regulation of protein and organelle delivery to and from synapses involves signal transduction cascades, and in particular alterations to effector protein phosphorylation via kinases and phosphatases [1, 2]. Interestingly, some of the kinases and phosphatases that regulate synaptic activity have now been shown to also regulate neuronal transport processes. For example, cdk5/p35 has roles in both pre- and post-synaptic function and in synaptic vesicle release, but also functions in regulating axonal transport [3–5]. GSK3β fulfils key synaptic functions linked to long-term potentiation (LTP) and long-term depression (LTD) [6, 7] but also phosphorylates kinesin-1 molecular motor to control release of cargoes from motor proteins [8]. In a similar fashion, extracellular signal-regulated kinases control the strength of synaptic transmission and also phosphorylate kinesin-1 motors to regulate axonal transport [9–11]. Finally, PP1 commands various aspects of synaptic and structural plasticity but targets kinesin-1 to control its function in transport [4, 12]. Such evidence suggests that the activities of kinase and phosphatase modulators of synaptic function are linked to their delivery to synapses via the control of axonal transport. This notion is in line with features of neurodegenerative diseases such as Alzheimer’s disease, Parkinson’s disease and amyotrophic lateral sclerosis/frontotemporal dementia (ALS/FTD) which display both synaptic dysfunction and damage to axonal transport [13–15]. One attractive possibility is that alterations to key kinases and phosphatases may underlie both synaptic and axonal transport damage. However, whilst the roles of kinases and phosphatases in modulating both synaptic and axonal transport functions are becoming clearer, as yet we know little of the mechanisms by which such enzymes and synaptic cargoes are transported through axons and delivered to synapses. Recently, progress has been made in this area with the discovery and characterisation of novel brain enriched kinases LMTKs. Here we describe this progress.

## The LMTK protein family

LMTKs are membrane associated serine/threonine protein kinases. The protein family consists of four members encoded by three genes LMTK1-a and LMTK1-b (encoded by *LMTK1* also known as *AATK* gene), LMTK2 (*LMTK2*) and LMTK3 (*LMTK3*) [16–21]. LMTK1-a and LMTK1-b are splice variants described in mouse while in human only the LMTK1-b homologue has been reported yet. LMTK1-a and LMTK1-b differ in their first 57 amino acids encoding exon which is missing from LMTK1-a. Due to parallel discoveries, LMTKs are known in the literature by several alternative names (Table 1) but to avoid confusion a new nomenclature has recently been introduced renaming the LMTK protein family to ‘lemur tail kinases’ [22, 23]. Some of the alternative names are especially misleading and they root in the finding that the amino acid sequences of LMTK kinase domains are highly similar in their catalytic sites to tyrosine kinases. This similarity led to the anticipation that LMTKs are dual-specific serine/threonine-tyrosine kinases, however, several studies have since demonstrated that LMTKs solely phosphorylate serine and threonine residues [19, 21, 24–26]. It also must be noted that because kinase classifications are based on the catalytic domain sequence similarities not on real targeted residues these classification studies with one exemption still categorise LMTKs among receptor tyrosine kinases [27–29]. However, we would like to emphasise again that according to our recent knowledge LMTKs are serine/threonine kinases.

**Table 1:** Alternative LMTK gene and protein names appearing in the literature

LMTK family member	Alternative names	First appearance in the literature
lemur tail kinase 1 (LMTK1, LMTK1-a and 1-b)	apoptosis-associated tyrosine kinase (AATK, AATYK, B-AATYK)	[17, 18, 203]
	apoptosis-associated tyrosine kinase-1 (AATYK1, AATYK1A and 1B, AATKA and AATKB)	[16, 27, 204]
	human AATYK short isoform-p35 binding polypeptide (hAATYKs-p35BP)	[61]
	KIAA0641	[40]
	lemur kinase 1 (LMR1)	[24, 28]
	lemur tyrosine kinase 1 (LMTK1, LMTK1A and 1B)	[47, 90]
lemur tail kinase 2 (LMTK2)	apoptosis-associated tyrosine kinase-2 (AATYK2)	[27]
	brain enriched kinase (BREK)	[21]
	Cdk5/p35-regulated kinase (Cprk)	[20]
	KIAA1079	[38]
	kinase/phosphatase/inhibitor-2 (KPI-2)	[19]
	lemur kinase 2 (LMR2)	[24, 28]
	lemur tyrosine kinase 2 (LMTK2)	[39]
lemur tail kinase 3 (LMTK3)	apoptosis-associated tyrosine kinase-3 (AATYK3)	[27]
	KIAA1883	[37]
	lemur kinase 3 (LMR3)	[24, 28]
	lemur tyrosine kinase 3 (LMTK3)	[47]

**Fig. 1:**
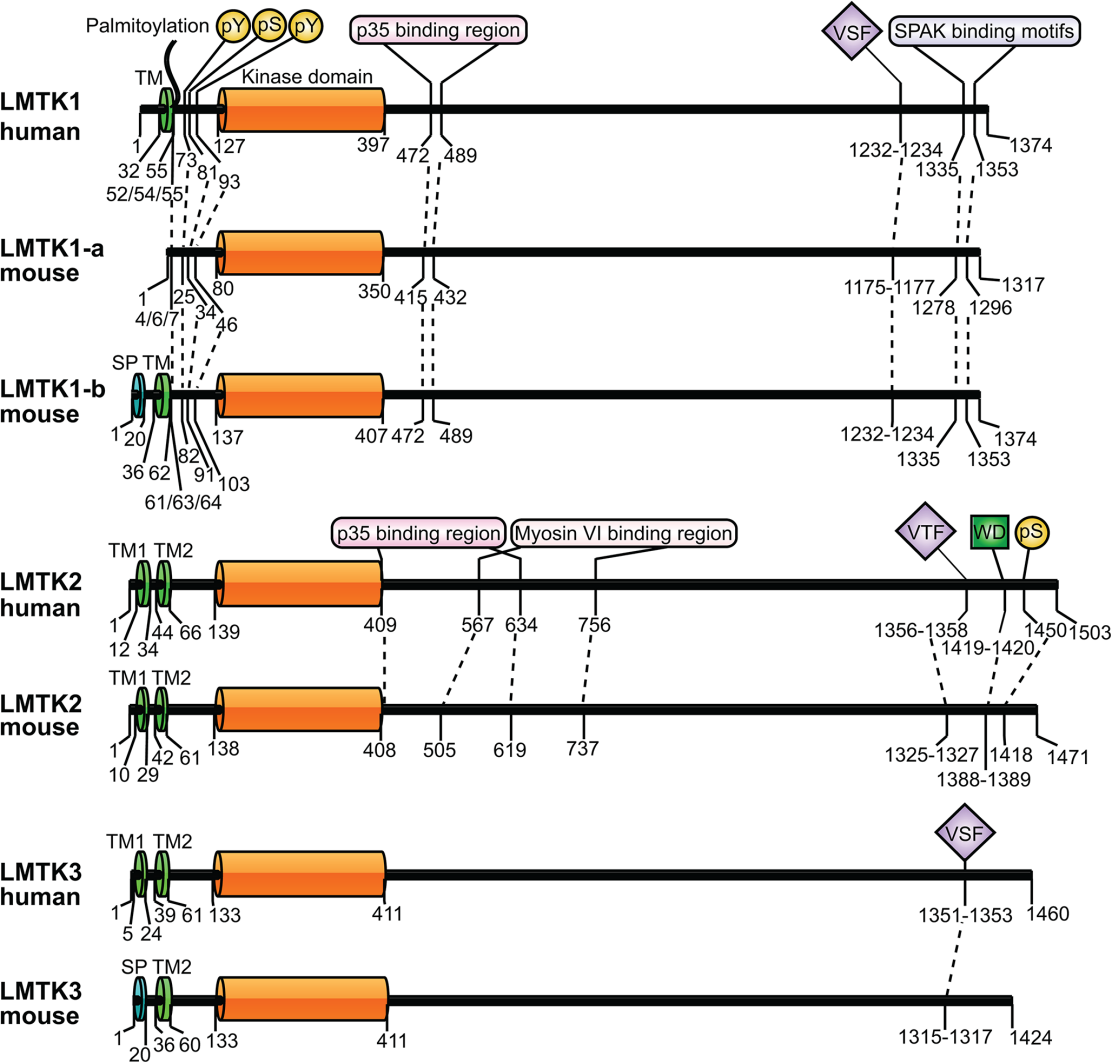
Domain organisation, protein interaction sites and post translational modifications of human and mouse LMTKs. LMTK1-b and mouse LMTK3 contain an amino-terminal signal peptide (SP) and a transmembrane (TM) domain while LMTK1, LMTK2 and human LMTK3 contain two TM domains. LMTK1-a and LMTK1-b are also associated with membranes by their three palmitoylated cysteine residues in their amino-termini. The membrane targeting regions are followed by a kinase domain and a long ‘tail’. The valine-serine-phenylalanine (VSF) and valine-threonine-phenylalanine (VTF) binding motifs of PP1C and the tryptophan-aspartic acid (WD) KLC binding motif are shown. Binding regions of LMTK interacting partners p35, myosin VI and SPAK are marked. Experimentally confirmed phosphorylated serine (pS) and tyrosine (pY) residues are shown. Numbers indicate amino acid positions. Dashed lines between LMTKs highlight homologous sites labelled in human LMTKs but present in mouse LMTKs. Protein domains were predicted using InterPro [218]

Protein domain predictions suggest that LMTKs consist of an amino-terminal signal peptide and a transmembrane domain in LMTK1-b, or two transmembrane domains in LMTK2 and LMTK3 followed by a kinase domain and a long carboxyl-terminal ‘tail’ (Fig. 1). This ‘tail’ inspired the now used name after the long-tailed Madagascan primates, the ring-tailed lemurs. Intracellular topological analysis of LMTK2 has revealed that its transmembrane domains anchor the protein into membranes in such a way that both its amino- and carboxyl-termini face towards the cytoplasm [30]. Similar topological studies have not been conducted with LMTK1 and LMTK3 to understand whether they anchored in membranes in a similar fashion. Despite the lack of the transmembrane domain, LMTK1-a is also associated with membranes via its palmitoylated cysteine-4, 6 and 7 residues [31]. Interestingly, adjacent to its transmembrane domain LMTK1-b is also palmitoylated. Its palmitoylated cysteine-61, 63, 64 residues are homologue amino acids of LMTK1-a cysteine-4, 6 and 7. Importantly, this palmitoylation is critical for LMTK1-b subcellular localisation as mutating these cysteine residues prevents LMTK1-b localisation into endosomes [32]. The N-terminal membrane-targeting region is followed by the serine/threonine kinase domain. LMTKs display autophosphorylation and phosphorylate target proteins in experimental conditions where no other kinases are present i.e. in in vitro kinase assays and peptide microarrays, therefore, they can be considered as constitutively active kinases [19, 21, 24–26, 33, 34]. The crystal structure of LMTK3 kinase domain was recently solved to 2.1 Å resolution [26]. The domain can take an inactive conformation where the tyrosine in the aspartic acid-tyrosine-glycine (DYG; residues 313–315) motif occupies the space of the adenine ring of ATP in the active site of the kinase domain. Phosphorylation of tyrosine-321, 325 and 326 in the activation loop can move the DYG motif out from the active site enabling ATP binding and hydrolysis. Further analysis of LMTK3 kinase domain in in vitro kinase assays pinpointed that arginine residues in the target peptide in position − 3 and/or -2 are critical for maximal phosphorylation efficiency while hydrophobic residues between positions − 3 and + 1 reduce phosphorylation efficiency. Based on these in vitro assays, LMTK3 substrate consensus sequence reads as (-4) L/K-R-R/X-X-S/T, where X is any residue [26]. Thus, LMTK3 is potentially an arginine directed serine/threonine kinase. This warrants the question whether LMTK1 and LMTK2 behave similarly and require arginine nearby the targeted serine or threonine residue. Finally, the long carboxyl-terminal regions of LMTKs contain several proline-rich so called PxxP motifs [17]. PxxP motifs can directly interact with sarcoma (Src) homology-3 (SH3) domains containing proteins [35, 36]. SH3 domains are present in numerous kinases or in their adaptor proteins, therefore, it is possible that LMTKs phosphorylate or phosphorylated by SH3 domain containing kinases, or function as scaffolding proteins for these kinases.

RNA and protein expression screens have clearly shown that LMTKs are present in most tissues but display the highest expression in neuronal tissue [16, 17, 19–21, 25, 37–40]. Although LMTKs show widespread protein expression across the brain their expression varies between different brain regions in mouse and rat. Thus, LMTK1 is more uniformly expressed across the brain while LMTK2 shows stronger expression in the cerebral cortex, hippocampus, thalamus, cerebellum and olfactory bulb. At the same time, LMTK3 displays the strongest immunoreactivity in the hippocampus, thalamus and cerebellum [16, 18, 20, 41, 42]. Expression profile analysis of LMTK1 and LMTK2 in developing brain and spinal cord has shown that LMTK expression increases gradually in early postnatal days suggesting that they might play role in neuronal development [20, 21, 32, 34, 42–44]. In neurons, LMTKs are found in all cellular compartments from the cell body through axons, dendrites and growth cones. Intracellularly, LMTKs are anchored into membranes such as in the Golgi apparatus, plasma membrane and various endosomal sub-compartments including early endosomes, recycling endosomes, pericentrosomal endosomal recycling compartment, and perinuclear endosomal recycling compartment [16, 20, 21, 31, 32, 41, 42, 45–52]. Some studies have also detected LMTK2 and LMTK3 in the nucleus in cancer cells [33, 55–58], however, other studies which have analysed LMTK subcellular localisation in neurons did not observe LMTKs in the nucleus [20, 21, 32, 47, 48, 51, 52, 59]. The reason why LMTKs locate in the nucleus in cancerous cells and not in neurons, and whether it is typical in all type of cancer and neuron is not known yet, and worth further investigation.

### LMTK2 and axonal transport

In neurons, long-range transport of various cargoes between the cell body and the synaptic region is mediated by kinesin and cytoplasmic dynein-1 motor proteins running along microtubule tracks. Short-range transport within the synaptic area, organisation of synaptic receptors and release of neurotransmitters depend on the actin cytoskeleton and myosin motors. Growing amount of evidence highlights that LMTKs function in fundamental transport events either via scaffolding motor proteins and cargoes or regulating signalling cascades involved in intracellular transport events (Table 2). Thus, LMTK2 interacts with the molecular motor proteins kinesin-1 and myosin VI, and also regulates kinesin-1-based transport [47, 48, 52, 60]. LMTKs also bind to a large number of proteins which place LMTKs centrally in signalling pathways associated with intracellular trafficking and synaptic communication including some key synaptic kinase, and phosphatase such as cdk5/p35, GSK3β and PP1 [19, 20, 58, 61]. Finally, all LMTKs are involved in endosomal vesicle transport either by binding to endosomal cargoes or regulators of endosomal trafficking [47, 48, 62–65]. Below we describe and critically discuss the role of LMTKs in intracellular trafficking events with a special focus on synaptic functions.

**Table 2:** LMTK binding partners

LMTK family member	Binding partner	Function of interaction	References
LMTK1	cdk5/p35^a^	Regulates LMTK1 activity and signalling	[61]
	PP1Cα^a^	Scaffolding to regulate NKCC1 activity	[68]
	SPAK	Scaffolding to regulate NKCC1 activity	[68, 205, 206]
	Src^a^	phosphorylates LMTK1; endosome trafficking	[31]
	TBC1D9B^a^	Regulates TBC1D9B activity and endosome trafficking	[63]
	TBC1D11^a^	Unknown; endosome trafficking?	[63]
LMTK2	AR	Regulates AR activity	[207]
	cdk5/p35^a^	Regulates LMTK2 activity and signalling, scaffolding to mediate axonal transport of cdk5/p35	[20, 60, 74]
	CFTR^a^	Regulates CFTR function and endocytosis	[24, 62]
	Inh2	Unknown	[19]
	KLC1^a^	Scaffolding to regulate axonal transport	[52]
	KLC2^a^	Scaffolding to regulate axonal transport	[52]
	Myosin VI^a^	Endosome trafficking	[47, 48]
	PP1Cα^a^	Regulates PP1C activity and signalling	[19, 60, 74]
LMTK3	α-adaptin^a^	Unknown; endosome trafficking?	[41]
	CDC37	Stabilising LMTK3 as a HSP90 co-chaperone	[26]
	DDX5	MicroRNA regulation	[56]
	ERα	Protects ERα from degradation	[33]
	GluA1^a^	Unknown; receptor internalisation?	[64]
	GRB2^a^	Scaffolding to regulate GRB2 downstream signalling	[57]
	KAP1	Scaffolding to regulate gene expression	[58]
	KCC2^a^	Regulates KCC2 activity	[51]
	Lamin A	Heterochromatin tethering to nuclear lamina	[58]
	NUSAP1^a^	Microtubule stabilisation	[85]
	PP1Cα^a^	Scaffolding to regulate KAP1 activity	[58, 69]

^a^LMTK binding partners involved in intracellular transport or linked to intracellular trafficking by LMTKs

AR androgen receptor, CDC37 cell division cycle 37, cdk5/p35 cyclin dependent kinase-5/p35, CFTR cystic fibrosis transmembrane conductance regulator, DDX5 aspartic acid-glutamic acid- alanine-aspartic acid (DEAD)-box RNA helicase p68, ERα oestrogen receptor alpha, GluA1 glutamate ionotropic receptor α-amino-3-hydroxy-5-methyl-4-isoxazolepropionic acid (AMPA)-type subunit-1, GRB2 growth factor receptor-bound protein-2, Inh2 protein phosphatase inhibitor-2, HSP90 heath shock protein 90, KAP1 Krüppel-associated box-associated protein-1, KCC2 potassium-chloride cotransporter 2, KLC1/2 kinesin-1 light chain-1 and 2, LMTK lemur tail kinase, NKCC1 Na-K-2Cl cotransporter-1, PP1Cα catalytic subunit of protein phosphatase-1 alpha, NUSAP1 nucleolar and spindle-associated protein 1, SPAK sterile 20 protein-related proline-alanine-rich kinase, Src sarcoma kinase, TBC1D9B transferrin receptor-like 2-budding uninhibited by benzimidazoles-2-cell division cycle protein-16 homolog-1 domain family member-9B, TBC1D11 TBC-1 domain family member-11

**Fig. 2:**
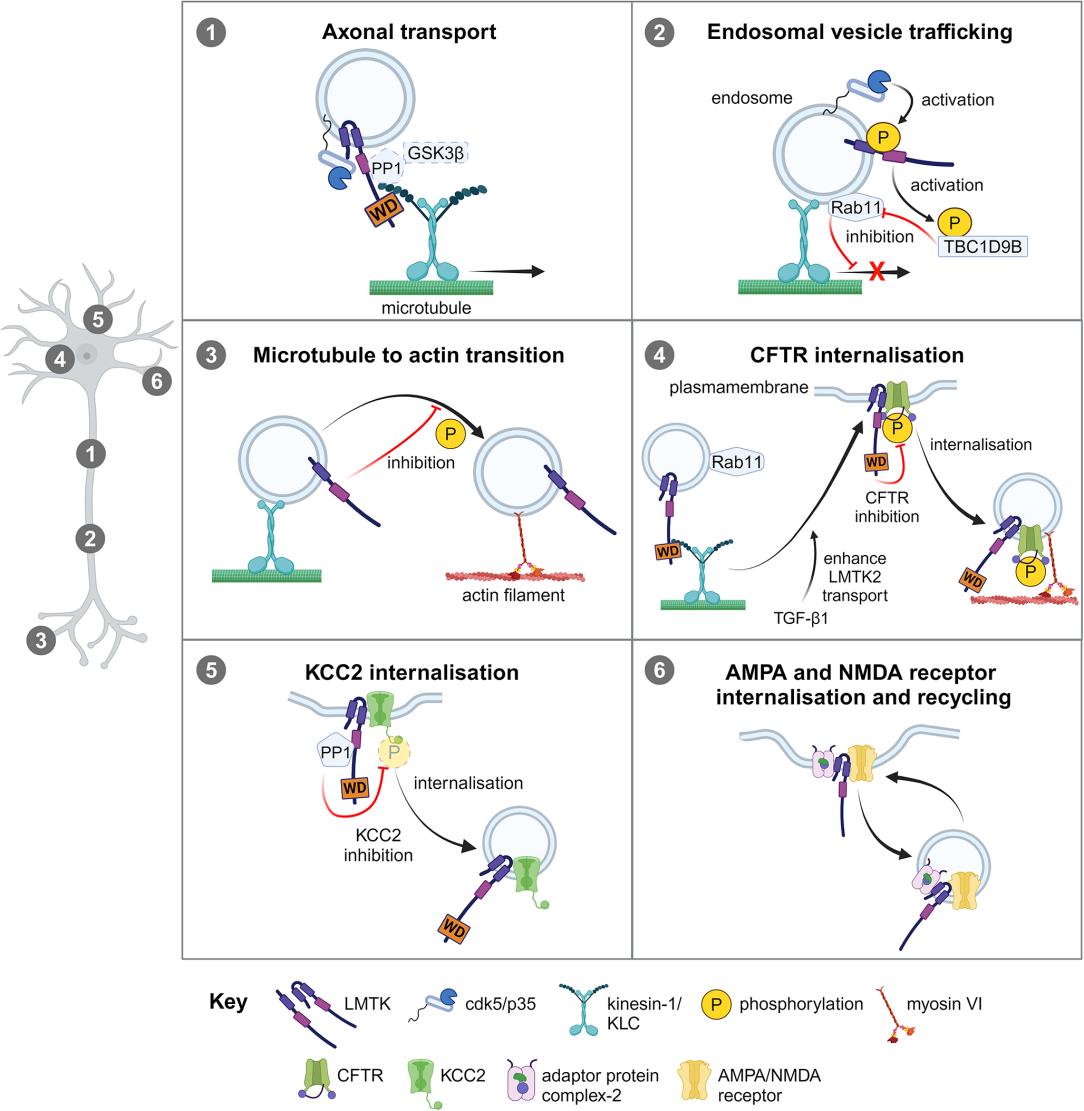
Mechanisms of intracellular transport regulated by LMTKs. (1) LMTK2 mediates axonal transport of cdk5/p35. LMTK2 scaffolds cdk5/p35 and kinesin-1-KLC via its tryptophan-aspartic acid (WD) motif in a complex to promote cdk5/p35 transport. It is possible that PP1 and GSK3β are also transported in this way towards the synapse. (2) LMTK1 regulates endosomal vesicle trafficking. cdk5/p35 phosphorylates LMTK1-a to activate it which then stimulates TBC1D9B. It in turn inhibits Rab11 by leaving it in a GDP bound inactive state leading to blocked transport of Rab11-positive endosomal vesicles. (3) LMTK1 controls microtubule-actin transition. LMTK1-a inhibits the transition of endosomes from microtubule tracks to actin cytoskeleton in neurite tips and it requires LMTK1 kinase activity. (4) LMTK2 promotes CFTR internalisation. TGF-β1 signalling enhances LMTK2 transport to the plasma membrane on Rab11-positive endosomes. In the plasma membrane, LMTK2 binds to CFTR to phosphorylate and inactivate it. Phosphorylated CFTR is internalised in an LMTK2 and myosin VI-dependent manner. (5) LMTK3 participates in KCC2 internalisation. LMTK3 scaffolds PP1 and KCC2 together to promote KCC2 dephosphorylation, inactivation and its internalisation. (6) LMTK3 is involved in AMPA and NMDA receptor internalisation and recycling. LMTK3 scaffolds together AMPA and NMDA receptor subunits with adaptor protein complex-2 and thus can regulate their internalisation on clathrin coated vesicles

Kinesin-1 is one of the major motor proteins delivering cargoes to the synapses [66]. A proportion of kinesin-1 is a hetero-tetramer comprising of two kinesin-1 heavy chains and two kinesin-1 light chains (KLC). Among KLCs the two best characterised are KLC1 and KLC2. A key function of KLCs is cargo binding and we have recently demonstrated that LMTK2 binds to KLC1 and KLC2 [52]. This interaction involves a tryptophan-glutamic acid/tryptophan-aspartic acid (WE/WD) motif in the carboxyl-terminus of LMTK2 and the tetratricopeptide repeat domains of KLCs. LMTK2-KLC binding recruits kinesin-1 into the complex and thus facilitates anterograde axonal transport of LMTK2. LMTK2 is an interacting partner of cdk5/p35, a neuronal kinase that involved in key synaptic functions, such as synaptic vesicle endo- and exocytosis, neurotransmitter receptor regulation, LTP and LDP [3, 20]. LMTK2 interacts with cdk5 by binding directly to p35, one of the activator subunits of cdk5. We have shown that LMTK2 recruits p35 to a complex containing KLC1 and that siRNA mediated loss of LMTK2 disrupts axonal transport of both p35 and cdk5 in living rat primary neurons [52]. Thus, LMTK2 acts as a scaffolding protein between kinesin-1 motor and a key neuronal cargo, cdk5/p35 (Fig. 2). Interestingly, siRNA knock down of LMTK2 did not completely block axonal transport of cdk5 and p35. One explanation to this is that other LMTKs can function as scaffolds between p35 and KLC. Indeed, sequence analysis revealed that both LMTK1 and LMTK3 contain conserved WE/WD motifs in their carboxyl-terminal tails. Furthermore, LMTK1 has also been identified as a binding partner of p35, and LMTK3 has been shown to be phosphorylated by cdk5/p35 [44, 45, 61]. Thus, potentially all LMTKs may function as scaffolding ligands between cdk5/p35 and KLCs. LMTK2 also binds to the catalytic subunit of PP1 (PP1C) via its valine-any residue-phenylalanine PP1C docking motif in its carboxyl-terminal tail [19]. PP1 is also strongly involved in the control of several synaptic function including α-amino-3-hydroxy-5-methyl-4-isoxazolepropionic acid (AMPA) and N-methyl-D-aspartic acid (NMDA) receptor signalling, and LTP and LTD induction [67]. LMTK2 not only binds to both cdk5/p35 and PP1 but also bring them together in one signalling cascade with GSK3β (see more details below); GSK3β is a kinase which also fulfils key synaptic functions linked to LTP and LTD [6, 7, 60]. Since LMTK2 scaffolds cdk5/p35 with kinesin-1 to transport it to synapses it is tempting to say that LMTK2 is also involved in axonal transport of other synaptic regulators such as PP1 and GSK3β as well. Clearly, details of this scenario are not known but one possibility is that they are transported individually on LMTK2-KLC-kinesin-1 complex. However, since the synaptic functions of cdk5/p35, PP1 and GSK3β are related, another attractive possibility is that all these synaptic players form a multiprotein complex for their axonal transport and delivered together towards synaptic terminals where LMTK2, as a major scaffold, links them to kinesin-1 motor. It also worths to note that LMTK1 and LMTK3 are also binding partners of PP1C and in this way they could be also involved in axonal transport of PP1 [58, 68, 69]. In conjunction with PP1 it is also important to mention that LMTK2 is associated with protein phosphatase inhibitor-2, one of the synaptic regulators of PP1, moreover, protein phosphatase inhibitor-2 is potentially regulated by GSK3β and cdk5/p35 [19, 67, 70–72]. Based on these findings, the above concept can be expanded that protein phosphatase inhibitor-2 might be also transported together with the LMTK2 scaffolded kinase-phosphatase complex and, additionally, LMTK2 might act as a regulatory scaffold to specifically control their activity during transport and within synapses. However, it is obvious that more work is needed to investigate all these possibilities and clarify what is the exact mechanism by which these synaptic proteins are transported through axons, whether these regulatory enzymes are transported in their active or inactive form and decipher how LMTK2 scaffolds them together.

Another intriguing question is how LMTK mediated axonal transport is regulated. GSK3β is a well-known regulator of axonal transport and it has been shown to phosphorylate KLC2 to reduce transport of vesicle and protein cargoes, and may also phosphorylate KLC1 [8, 60, 73]. We have identified an LMTK2-associated signalling cascade regulating GSK3β kinase activity and thus KLC2 cargo binding [60, 74]. In this signalling cascade, cdk5/p35 increases LMTK2 activity by phosphorylating it on serine-1418. Activated LMTK2 in turn binds to and induces phosphorylation of PP1C on threonine-320 to attenuate its activity. This inhibition of PP1C leads to increased phosphorylation of GSK3β on serine-9 which blocks its kinase activity. Inhibited GSK3β is not able to phosphorylate KLC2, efficiently promoting KLC2 binding to its cargo, suppressor of mothers against decapentaplegic 2 (Smad2). Thus, LMTK2 functions as a negative regulator of GSK3β activity to control axonal transport [60, 75–78]. Apart from GSK3β, other protein kinases also have been shown to regulate axonal transport including Akt and extracellular signal-regulated kinases, and, although LMTKs are implicated in the control of these kinases further extensive work is needed to clarify whether the link between LMTKs and these kinases plays role in the governance of axonal trafficking [1, 33, 57, 79–84].

LMTKs are also implicated in the regulation of microtubule cytoskeleton which serves as tracks for kinesin and dynein motor proteins, and signals affecting microtubule organisation and remodelling can highly influence axonal transport. LMTK1 has been shown to be involved in microtubule reorganisation in neuron-like Neuro2a cells since a kinase-deficient mutant of LMTK1-a increases microtubule density in the cell periphery and decreases it in the perinuclear area [50]. Similarly, loss or inhibition of LMTK2, or LMTK3 in cell lines leads to perturbed microtubule distribution and organisation [78, 82, 85, 86]. The exact mechanism by which LMTKs induce microtubule remodelling is not known but one possibility is, at least in the case of LMTK3, that it is linked to altered expression of proteins related to microtubule organisation. Particularly, LMTK3 has been shown to bind to nucleolar and spindle associated protein 1 (NUSAP1), a regulator of microtubule organisation [85]. This interaction potentially stabilises NUSAP1 as treating cells with C28, an LMTK3 inhibitor which induces LMTK3 instability and degradation by the proteasome, or siRNA silencing of LMTK3 reduces NUSAP1 protein expression [26, 85]. Moreover, LMTK3 inhibition and knockdown also reduces the expression of the NUSAP1 regulated kinase cdk1, and decreases phosphorylation of the cdk1 target βIII tubulin, whose phosphorylation influences microtubule dynamics [85]. However, while these alterations potentially can cause microtubule instability further detailed investigations are needed to determine functional consequences of these affects in neurons and in the central nervous system with special focus on axonal transport.

### LMTKs and endosomal vesicle trafficking

The amount of synaptic receptors in the plasma membrane, axonal and dendritic outgrowth, dendritic arborisation and spine formation are critical for proper synaptic functioning and these processes are controlled by endosomal vesicle trafficking [87, 88]. A number of lines of evidence strongly link LMTKs to these processes. Firstly, LMTKs colocalise with several endocytic vesicle markers including Ras-analogue in brain (Rab) proteins, small GTPases which have important role in the organisation and regulation of endocytic vesicle trafficking and synaptic transmission. Thus, LMTK1-a and LMTK1-b colocalise with Rab5 and Rab11, LMTK2 is seen together with early endosome antigen-1, Rab5 and Rab11, while LMTK3 is present in the early endosome antigen-1-positive fraction following biochemical fractionation of brain lysates, and LMTK3 function is also associated with Rab14 activity [31, 32, 41, 46–48]. Since Rab5 is localised in early endosomes, Rab11 in slow recycling endosomes and Rab14 in recycling endosomes the above data strongly indicate that some of LMTKs functions is linked to early and recycling endosomes but not late endosome/lysosome compartments or the secretory pathway [53, 54].

Secondly, it has been demonstrated in a set of papers from the Hisanaga group that LMTK1 controls Rab11-positive endosomal trafficking to make possible proper dendritic spine formation, maturation and density, axonal and dendritic outgrowth, and dendrite branching [31, 32, 45, 46, 49, 50, 63, 89, 90]. All these processes are controlled by a novel signalling cascade in which LMTK1 is centrally placed as a negative regulator of Rab11 activity and endosome transport (Fig. 2) [63]. In this pathway, cdk5/p35 phosphorylates LMTK1-a on serine-34 to increase its activity. LMTK1-a in turn binds to and activates transferrin receptor-like 2-budding uninhibited by benzimidazoles-2-cell division cycle protein-16 homolog-1 domain family member-9B (TBC1D9B), a GTPase-activating protein of Rab11. Finally, increased GTPase activating function of TBC1D9B leads to blocked Rab11 activity probably by returning Rab11 into an inactive GDP-bound state and in this way inhibiting endosome trafficking [63]. The activating effect of LMTK1-a on TBC1D9B is linked to its kinase activity, however, whether LMTK1-a phosphorylates TBC1D9B directly or indirectly needs to be elucidated. Another regulatory pathway for endosomal trafficking also potentially involves LMTK1 and cdk5/p35 together with Src and Fyn, two non-receptor tyrosine-protein kinases which regulate endosomal trafficking, including Rab11-positive endosomes, to the plasma membrane [91, 92]. LMTK1-a has been shown to interact with Src and Fyn when LMTK1-a is localised in endosome membranes [31]. The exact details of their association are not known but one possibility is that the SH3 domains of Src and Fyn bind to the PxxP motifs of LMTK1. It has been demonstrated that both Src and Fyn phosphorylate LMTK1-a potentially on tyrosine-25 and 46. Interestingly, LMTK1-a tyrosine phosphorylation is inhibited when cdk5/p35 phosphorylates it on serine-34 [31, 45]. Since, as we described it above, LMTK1 phosphorylation on serine-34 by cdk5/p35 induces a signalling cascade involving TBC1D9B to control Rab11-positive endosomal trafficking it is an exciting question to follow how Src and Fyn mediated LMTK1 phosphorylation integrates into this process. Obviously, further research is needed to elucidate all aspects of this phosphorylation cascade and especially the functional consequences of LMTK1 tyrosine-25 and 46 phosphorylation on endosome transport, and their influence on synaptic functions.

Thirdly, LMTK2 interacts with myosin VI, an unconventional myosin playing role in synapse formation, synaptic receptor trafficking, LTD induction, synaptic vesicle resupply and endosome sorting [47, 48, 93–98]. LMTK2 binds with a region close to its kinase domain to the tryptophan-tryptophan-tyrosine cargo binding motif of myosin VI to colocalise in early and recycling endosomes [48, 99]. LMTK2 and myosin VI siRNA knockdown experiments have demonstrated that they function together in the delivery of cargoes from early endosomes to the endocytic recycling compartment [47, 48]. Their associated role in trafficking of important synaptic regulators is further supported by the findings that both LMTK2 and myosin VI are interacting partners of cystic fibrosis transmembrane conductance regulator (CFTR), a cyclic adenosine monophosphate-activated chloride ion channel [24, 62, 98]. CFTR is predominantly expressed in epithelial cells but it is also present in the central nervous system where it modulates motor neuron resting potential and glycine/gamma-aminobutyric acid (GABA) receptor mediated synaptic depolarisation [100–106]. LMTK2 has been shown to phosphorylate CFTR on serine-737 which inhibits its ion channel function and enhances its endocytosis [24, 62, 107, 108]. LMTK2 mediated CFTR phosphorylation leans on transforming growth factor-β1 signalling which increases Rab11-dependent transport of LMTK2 to the cell membrane in order to phosphorylate CFTR and boost its endocytosis (Fig. 2) [109]. The above data suggest that LMTK2 and myosin VI might function together in endosomal trafficking of CFTR, however, it requires further investigation to formally confirm it, to understand whether LMTK2 recruits myosin VI to endosomes or other way around, and to decipher whether LMTK2 phosphorylates myosin VI to regulate intracellular transport.

Fourthly, LMTK3 has recently been identified as a potassium-chloride cotransporter 2 (KCC2) interacting partner and regulator in the plasma membrane [51, 110]. KCC2, like CFTR, is a regulator of chloride ion homeostasis in neurons and its activity is mandatory for glycine/GABA receptor mediated fast synaptic inhibition [111]. Experiments utilising acute hippocampal slices treated with the LMTK3 inhibitor C28 or isolated from LMTK3 knockout mice have revealed that the presence and activity of LMTK3 is necessary for KCC2 controlled chloride ion extrusion, GABA-induced currents, and neuronal excitability [26, 51]. KCC2 activity is inhibited and its internalisation promoted by dephosphorylation of its serine-940 residue [112]. It has been shown that LMTK3-KCC2 interaction is required to recruit PP1 to KCC2 to modulate its serine-940 dephosphorylation [51, 113]. Thus, LMTK3 diminishes KCC2 activity by reducing its phosphorylation on serine-940 via scaffolding it with PP1, and although it has not been directly tested it potentially promotes its endocytosis similar to LMTK2 and CFTR.

Fifthly, LMTK3 is also targeted into AMPA and NMDA receptor-positive endosomes, and involved in their internalisation and recycling (Fig. 2) [41, 64, 114]. Particularly, LMTK3 knockout in mice increases intracellular accumulation of glutamate ionotropic receptor AMPA-type subunit-1 (GluA1), and glutamate ionotropic receptor NMDA-type subunit-1 and 2B (GluN1 and GluN2B) implying that LMTK3 can control receptor trafficking [41, 64]. AMPA and NMDA receptors are internalised via clathrin-coated vesicles in an adaptor protein complex-2-dependent manner [115–119]. Adaptor protein complex-2 is a hetero-tetramer and LMTK3 has been shown to interact with its α-adaptin subunit, a subunit involved in the recruitment of endocytic accessory proteins [41, 64, 120]. Endocytic accessory proteins have role in cargo recognition and scaffolding raising the possibility that LMTK3 is a novel accessory protein for AMPA and NMDA receptor subunits. LMTK3 has also been identified as a modulator of Rab14-linked endosomal trafficking [65]. Rab14 is one of the several Rabs enriched on synaptic vesicles and is also seen on clathrin-coated vesicles [121, 122]. In this pathway, LMTK3 mediates phosphorylation of Rab-coupling protein, a modulator of Rab14, on serine-435. This phosphorylation induces Rab-coupling protein association with Rab14 and promotes Rab14-dependent endosomal trafficking [65]. However, the involvement of LMTK3 on Rab14-associated endosomal trafficking was analysed in non-neuronal cells, therefore, more work is required to unveil whether LMTK3 regulates Rab14-positive synaptic vesicle trafficking and to decipher the exact mechanisms by which LMTK3 controls these processes.

Finally, LMTK1 is also implicated in processes controlling endosomal switch from the microtubule tracks to actin filaments making possible for endosomes to approach the plasma membrane and fuse with it. LMTK1-a has been shown to changes microtubule-actin track together with endosomes and this action is regulated by its own kinase activity since wild-type LMTK1-a localises predominantly on microtubules, and do not colocalize with actin filaments. In contrast, kinase deficient mutant LMTK1-a partially colocalises with actin filaments in the neurites of neuron-like Neruro2a and PC12 cells [50]. These data suggest that active LMTK1-a can control endosomal vesicle transition onto actin filaments and inhibition of LMTK1-a is necessary to transport recycling endosomes to the distal tips of neurites (Fig. 2). The precise details of LMTK-dependent microtubule-actin transition of endosomes is not known but since specific effectors can regulate cytoskeletal affinity of endosomes LMTK1-a might be such an effector for Rab11. There is now also evidence that LMTK3 regulates actin cytoskeleton remodelling [57]. This mechanism requires LMTK3 to form a complex with growth factor receptor-bound protein-2 and son of sevenless homolog-1 to activate Ras which in turn switches on cell division control protein-42 homolog. It then activates serum response factor, a transcription factor for the genes encoding integrin subunits α5 and β1. Intracellularly, integrins are linked to the actin cytoskeleton via adaptor proteins and can actively rearrange the cytoskeleton [57]. Integrins are involved in the control of vesicular trafficking and connect cells to cells [123], therefore, from a neuronal perspective, LMTK3 regulated integrins and actin cytoskeleton might also have important role in the arrangement of correct synaptic structures, synaptic vesicle organisation, synaptic plasticity, and neurite outgrowth. However, it is important to note that the above outlined effect of LMTK3 on integrin expression was described in non-neuronal cell lines, therefore, more work is necessary to examine the role of LMTK3 on synapse formation and neuronal plasticity in conjunction with integrins and the actin cytoskeleton.

## LMTKs and neurodegenerative diseases

Loss of synapses is a defining feature in neurodegenerative diseases including Alzheimer’s disease, Parkinson’s disease and ALS/FTD. Since neurons are highly polarised cells with axons and dendrites, proper synaptic functioning requires a perfectly organised transport system. This special architecture and its dependence on transport events make neurons vulnerable against perturbations of intracellular transport. Damages to intracellular transport are very early defects in major neurodegenerative diseases such as Alzheimer’s disease, Parkinson’s disease, and ALS/FTD and a number of lines of evidence suggest that defective transport contributes to the pathogenesis of these disorders [13, 124]. As discussed above, LMTKs regulate axonal transport and endosomal vesicle trafficking, and involved in synaptic functions. There is now growing evidence that LMTK expression is decreased in many major neurodegenerative diseases (Table 3). Notably, decreased LMTK1 and LMTK2 protein expressions have been detected in the cortex and hippocampus of Alzheimer’s disease patients [52, 59, 125]. Interestingly, LMTK2 expression changes differently in affected (frontal cortex) and non-affected (cerebellum) brain regions in Alzheimer’s disease post-mortem brains. Immunoblot analysis of tissue lysates has revealed significantly reduced LMTK2 levels in mid dementia (Braak stage III-IV) and sever dementia (Braak stage VI) cases in the cortex compared to controls. In contrast, LMTK2 levels did not differ in Braak stage III-IV from control but were elevated in Braak stage VI in the cerebellum [52]. The early loss of LMTK2 in Braak stage III-IV cortical samples suggests that LMTK2 deficit might be an early pathogenic feature which are generally believed to contribute the most to the disease pathogenesis. Whether increased LMTK2 protein levels in the cerebellum in Braak stage VI represent neuroprotective effect is not clear. Additionally to their expression changes, LMTK2 has recently been predicted as an Alzheimer’s disease risk gene by computational analysis of genome-wide association studies while LMTK1 has been identified as a haplotype-associated risk factor of ALS/FTD and de novo copy number variation of LMTK1 in sporadic ALS also has been reported [126–128]. There are many further studies which analysed LMTK expression at RNA levels in patient tissues and in animal models of major neurodegenerative diseases including Alzheimer’s disease ALS/FTD, Huntington’s disease, and vascular dementia and the majority of these works also found decreased LMTK expression in disease affected brain regions (Table 3). Although the mechanism by which LMTK expression is deregulated in neurodegeneration is not fully known, recent experimental evidence indicates that amyloid-beta 1–42 peptide, which is deposited in Alzheimer’s disease brain forming a hallmark pathology, is potentially involved in it. It has been shown that amyloid-beta 1–42 perturbs the function of the translation regulator and *LMTK2* mRNA binding partner fragile X mental retardation protein leading to reduced *LMTK2* mRNA translation [129]. Another possible mechanism deregulating LMTK2 expression involves *LMTK2* mRNA methylation, known as N^6^-methyladenosine or m^6^A modification. This modification is essential for *LMTK2* mRNA translation as knocking out methyltransferase-like 3 and 14, enzymes involved in this modification process, in mice leads to significantly reduced LMTK2 expression [130]. Reduced methyltransferase-like 3 and 14 expression and concomitant diminished m^6^A modification has been reported in many neurodegenerative diseases including Alzheimer’s disease and ALS/FTD which could explain reduced LMTK2 expression in these disorders although further work is needed to confirm this possibility [131–133]. Some data also suggest that micro RNAs (miRNAs) can contribute to LMTK deregulation in neurodegenerative diseases. Specifically, expression of miR-34a, miR-182-5p and miR-338, miRNAs which regulate LMTK1 and LMTK3 production, is altered in Alzheimer’s disease, ALS/FTD and Huntington’s disease patients [134–147]. miR-338 is transcribed from intron seven of *LMTK1*, expressed in neuronal tissue and reduces the mRNA level of its own host gene *LMTK1* in B35 rat neuroblastoma cell line [148–153]. Similarly, miR-34a and miR-182-5p downregulate *LMTK3* mRNA and protein expression [56, 154]. Interestingly, LMTK3 can itself regulate miR-34a-5p and miR-182-5p expression, and this effect is probably linked to the interaction of LMTK3 with the DEAD-box RNA helicase p68 (also known as DDX5), an RNA binding protein involved in miRNA processing [56]. Further regulatory mechanisms such CpG island hypermethylation and 12-O-tetradecanoylphorbol-13-acetate/TPA-responsive element activation also known to control LMTK1 and LMTK2 expression, respectively [155–158]. Although these latter results are valuable it is not known whether they are involved in LMTK deregulation in neurodegenerative diseases, therefore, future studies are needed to explore this question in more detail.

**Table 3:** Alterations in LMTK expression in neurodegenerative diseases and in their model systems

Disease	LMTK family member	Sample type	Assay type	LMTK down / upregulation	References
Alzheimer’s disease	LMTK1	Post-mortem entorhinal cortex	DNA microarray	Down	[208]
		Post-mortem hippocampus	DNA microarray	Up	[209, 210]
		Post-mortem frontal cortex	IB	Down	[125]
	LMTK2	Post-mortem middle temporal gyrus, posterior cingulate cortex and entorhinal cortex	DNA microarray	Down	[211, 212]
		Post-mortem hippocampus	DNA microarray	Down	[210, 213]
		Tau P301L mutant mice cerebral cortex, hippocampus and cerebellum	RNAseq, DNA microarray	Down (cortex, hippocampus) Up (cerebellum)	[214]
		Post-mortem frontal cortex and cerebellum	IB	Down (cortex) Up (cerebellum)	[52]
		Post-mortem middle frontal gyrus and anterior hippocampus	IHC	Down	[59, 194]
		Amyloid-beta treated rat primary cortical neurons	RNAseq	Down	[129]
		Amyloid-beta treated PC12 cells	qPCR, IB	Down	[191]
	LMTK3	Tau P301L mutant mice cerebral cortex, hippocampus and cerebellum	RNAseq, DNA microarray	Down (cortex, hippocampus) Up (cerebellum)	[214]
ALS/FTD	LMTK1	Post-mortem temporal cortex	Genotyping and SNP microarray	Down	[126]
		SOD1 G93A mice spinal cord microglia	RNAseq	Down	[215]
		SOD1 G93A mice spinal cord astrocytes	DNA microarray	Down	[216]
		Leukocytes from patient blood	qPCR	Up	[134]
Huntington’s disease	LMTK2	D9-N171-98Q mouse striatum	DNA microarray	Down	[217]
Vascular dementia	LMTK2	Post-mortem hippocampus	DNA microarray	Down	[213]

ALS/FTD amyotrophic lateral sclerosis/frontotemporal dementia, IB immunoblot, IHC immunohistochemistry, LMTK1, 2, 3 lemur tail kinase-1, 2 and 3, qPCR quantitative polymerase chain reaction, RNAseq RNA sequencing, SNP microarray single nucleotide polymorphism microarray, SOD1 G93A mutant superoxide dismutase-1 in which glycine in position 93 is substituted by alanine

**Fig. 3:**
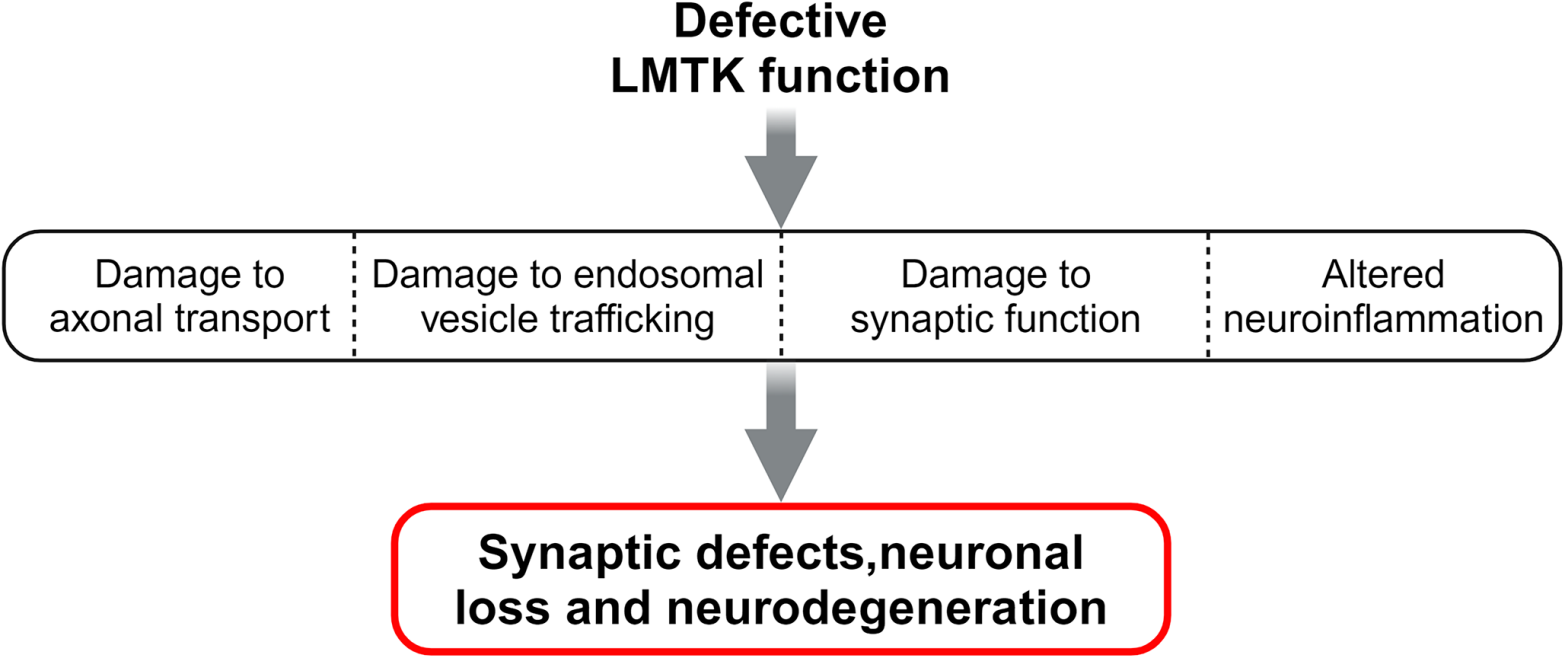
Defective LMTK function provides a mechanism by which many pathological features in neurodegenerative diseases might arise

A number of lines of evidence suggest that LMTK loss and thus their disfunction can potentially contribute to several pathological defects including transport and synaptic defects in neurodegenerative diseases (Fig. 3). Firstly, damage to axonal transport is an early feature in major neurodegenerative diseases and loss of synapses is seen in all these diseases. Axonal transport is critical for synaptic function because this is the way how synaptic vesicles and enzymes, assembled in the cell body, delivered to synaptic terminals. Cdk5/p35 is a key enzyme in the synapse and LMTK2 is essential for its transport [52]. Abnormal accumulation of cdk5/p35 in neuronal cell body have been observed in affected brain regions in post-mortem Alzheimer’s disease brains which is consistent with disrupted cdk5/p35 transport caused by loss of LMTK2 [159–162]. Perturbed cdk5/p35 transport can lead to defective synaptic functions seen in neurodegenerative diseases by deregulating cdk5-dependent pre- and postsynaptic processes such as neurotransmitter release, control of postsynaptic neurotransmitter receptor expression and clustering, and modulation of dendritic spine morphogenesis [3]. Assuming that PP1 and GSK3β are also transported to the synapse by the LMTK2-KLC-kinesin-1 motor complex, LMTK2 deficiency can induce further synaptic dysfunction in neurodegenerative diseases by disrupting axonal transport of these important synaptic signalling molecules. Likewise, loss of LMTK2 could also affect synaptic function via its interaction with myosin VI since myosin VI is known to play roles in synapse formation and synaptic receptor trafficking, and altered LMTK2 expression can lead to deleterious changes in these synaptic mechanisms [95–97]. Additionally, loss of LMTK2 can potentially perturb axonal transport governing signalling processes as well. GSK3β controls intracellular transport by phosphorylating molecular motors, adaptor proteins and cargoes, and, as we discussed it earlier, LMTK2 is a negative regulator of GSK3β [1, 60, 75–78]. In line with this, we have shown that siRNA loss of LMTK2 releases Smad2, a known kinesin-1 cargo, from KLC2 and this is mediated by GSK3β [60]. Increased GSK3β activity and concomitant disruption of transport have been reported in neurodegenerative diseases [73, 163–169] suggesting that LMTK deficiency can potentially perturb GSK3β-associated signalling cascades regulating axonal transport. Loss of LMTK can also contribute to synaptic defects in neurodegenerative diseases by affecting endosomal trafficking. It has been shown that axonal and dendritic transport of Rab11 is altered in primary neurons obtained from LMTK1 knockout mice, and in LMTK1 siRNA treated primary cortical neurons. Live imaging experiments have revealed abnormal anterograde bias in both axonal and dendritic transport of Rab11-positive endosomes. These endosomes are more dynamic in knockout cells and, specifically, they are characterised by increased anterograde run length and velocity in both axons and dendrites leading to abnormal neurite structures and synapse formation [49, 63, 90]. Loss of LMTK3 also affects endosomal transport and thus damages synaptic plasticity and LTP probably by disrupting endocytic trafficking of the AMPA and NMDA receptor subunits GluA1, GluN1 and GluN2B [41, 64]. LMTK3 knockout significantly increases intracellular localisation of these receptors in primary cortical neurons by either promoting their internalisation or reducing their export to the plasma membrane. This can lead to imbalanced receptor subunit composition and receptor distribution in neurodegenerative diseases. The detailed mechanism underlying this defect is not known yet, but one possibility roots in the fact that endosomal trafficking of these receptors is controlled by GSK3β and reduced LMTK expression increases GSK3β activity [60, 170, 171]. Another possibility is that LMTK3 loss directly disrupts organisation of clathrin-associated endocytic machinery since LMTK3 has been shown to bind to and potentially scaffolds together GluA1, and the adaptor protein complex-2 α-adaptin subunit, an important player of the endocytic machinery [41, 64].

Secondly, LMTK1 is involved in the endosomal transport of amyloid precursor protein (APP), a key axonal transport cargo in Alzheimer’s disease and whose mutations cause some familial forms of the disease. Proteolytic processing of APP by beta-site APP cleaving enzyme-1 (BACE1) and gamma-secretase generates amyloid-beta peptide [172]. Some roots of APP and BACE1 transport involves Rab11, and it has recently been shown that LMTK1-a co-localises with BACE1 in Rab11-positive recycling endosomes [125, 173–176]. Immunofluorescent localisation experiments confirmed that LMTK1-a controls subcellular localisation of BACE1 as LMTK1 knockdown disperses BACE1 from Rab11-positive recycling endosomes along the cytoplasm. Together with LMTK1-associated BACE1 delocalisation the amyloidogenic processing of APP is also significantly increases. However, the amount of non-amyloidogenic APP cleavage products are also show some increase in this experimental setup. Based on these findings, Komaki and colleagues speculated that LMTK1 not directly affects BACE1 mediated APP cleavage but rather regulates endosomal sorting of APP cleavage products [125]. It has recently been shown that affects changing APP distribution among different endosomal compartments and altering its endosomal trafficking influence amyloidogenic processing of APP [177]. Importantly, many further studies have demonstrated that disruption of APP transport is an early feature in Alzheimer’s disease and it promotes amyloidogenic processing of APP, moreover, altered processing of APP itself disrupts axonal transport [178–182]. These findings suggest that decreased LMTK1 expression in Alzheimer’s disease could contribute to amyloidogenic cleavage of APP and amyloid beta accumulation by perturbing APP endosomal trafficking which in turn can induce further transport and synaptic damages.

Thirdly, neuroinflammation is also seen in all major neurodegenerative diseases and recent evidence supports such notion that LMTK2 itself is linked to inflammation. Microglia activation is key for the development of neuroinflammatory processes and LMTK2 has been shown to regulate inflammatory actions in the microglia-derived BV-2 cell line [183–185]. Overexpression of LMTK2 promotes production of the anti-inflammatory cytokine interleukine-10 and reduces the amount of the pro-inflammatory factors interleukine-1β, interleukine-6 and tumour necrosis factor α in BV-2 cells in a lipopolysaccharide inflammation model [185]. There is also evidence that LMTK signalling partners cdk5, PP1 and GSK3β play role in microglia activation and neuroinflammation, and some of these effects are linked to nuclear factor erythroid 2-related factor 2 (Nrf2) and nuclear factor-kappa B (NF-κB), proteins associated with inflammatory signalling pathways [186–189]. LMTK2 has been shown to promote Nrf2 expression and anti-inflammatory signalling potentially by inhibiting GSK3β [185, 190, 191]. Therefore, loss of LMTK2 can contribute to microglia activation and neuroinflammation in neurodegenerative diseases by activating GSK3β and blocking the anti-inflammatory effect of Nrf2. LMTK2 also induces the activity of the pro-inflammatory factor NF-κB via the PP1-GSK3β-p65 pathway [75]. It is interesting that LMTK2 can induce both pro- and anti-inflammatory signalling cascades but it is also important to note that the effect of LMTK2 on NF-κB was tested in some cancer cell lines and not in microglia. Therefore, more research is needed to elucidate the role of LMTK2 in NF-κB-associated pro-inflammatory signalling pathways in microglia. It also worth further investigation to determine how these LMTK2-associated inflammatory signalling cascades in microglia contribute to neuroinflammation in neurodegenerative diseases.

Fourthly, LMTK2 can be involved in the microtubule-associated protein tau hyperphosphorylation in Alzheimer’s disease and other tauopathies. Hyperphosphorylated and misfolded tau forms intracellular neurofibrillary tangles, one of the hallmark pathologies in these diseases. Phosphorylation of tau blocks its microtubule binding and stabilising role which can cause axonal transport defects [192, 193]. GSK3β and cdk5 are two major tau kinases whose activity is increased and prolonged in Alzheimer’s disease, however, it is also known that cdk5 is a negative regulator of GSK3β and inhibits its activity by increasing its phosphorylation on serine-9 [4, 60, 74, 192]. This virtual contradiction can be explained by the decreased LMTK2 expression in Alzheimer’s disease [52, 59]. Since LMTK2 is a molecular brake between cdk5 and GSK3β [60, 74] its loss in the disease can lead to increased GSK3β activation and concomitant abnormal tau hyperphosphorylation which then can efficiently damage microtubule tracks. Supporting this notion, a recent study has found negative correlation between reduced LMTK2 levels and increased tau phosphorylation in Alzheimer’s disease [194], however, the direct mechanistic link between loss of LMTK2, increased GSK3β activity and tau hyperphosphorylation requires further investigation to confirm this concept.

Finally, *LMTK* knockout animal models strengthen further the notion that LMTKs play crucial role in synaptic functions and their loss can contribute to diseases affecting the nervous system [41, 44, 64, 90]. *LMTK1* knockout does not cause any apparent gross anatomical abnormalities in the brain but significantly increases the number of excitatory synapses throughout the whole brain. Detailed behavioural tests have shown hyperactivity, high motor coordination, less anxiety and anti-depressant behaviour in these animals [44]. Physical, behavioural and cognitive examinations of *LMTK3* knockout mice have revealed numerous neurological abnormalities, some of them similar to those seen in *LMTK1* knockout animals, including locomotor hyperactivity, reduced anxiety, hyper-sociability, depression-like behaviour, and defective memory and learning together with increased dopamine metabolism in the striatum [41, 64]. Additionally, electrophysiological analysis of field excitatory post-synaptic potential in the hippocampus showed impaired LTP in *LMTK3* knockout mice [64]. Interestingly, some of these defects resemble symptoms associated with psychiatric disorders such as schizophrenia and bipolar disease and treating LMTK3 knockout mice with clozapine, an antipsychotic drug used for schizophrenia treatment, suppressed behavioural abnormalities and improved memory and learning deficits [64]. These data suggest that LMTK1 and LMTK3 play important role in synaptic activity, learning and memory supporting their importance in neurodegenerative diseases. At the same time, effects of LMTK2 loss in the nervous system were not properly tested and reported in LMTK2 knockout animals [39]. To dissect neuronal function of LMTK2, this animal model needs to be further investigated or novel models need to be developed.

## Conclusions

Recently emerging evidence has now substantially widened our understanding and strengthened the concept that LMTKs have important role in several intracellular transport events. These functions are mediated in two ways; firstly, via the kinase activities of the LMTKs and secondly, via their interaction with key transport and regulatory proteins. Thus, LMTK members bind to and regulate cdk5/p35, GSK3β and PP1 activities via their kinase activities but also interact directly with two major molecular motors, kinesin-1 and myosin VI. The LMTKs therefore combine a scaffolding function that enables the intracellular transport of key signalling molecules to their appropriate destinations including the synapse, with the ability to regulate the activities of cdk5/p35, GSK3β and PP1 via their kinase activities. LMTKs also mediate endosomal transport to regulate neurite outgrowth, dendrite branching and dendritic spine formation, and control pre- and postsynaptic receptor and vesicle trafficking, and neuronal excitability. However, despite of all these experimental data the picture is still not clear. There are several open questions to be answered by future studies to decipher the exact functions of LMTKs in intracellular transport and synaptic functions. LMTKs share many binding partners and contain similar protein interaction motifs such as the WE/WD motif, additionally all of them can be found in endosomes. Does it mean functional redundancy among them? Or rather their spatiotemporal distribution is tightly controlled and they are never targeted into the same subcellular compartment in the same time? What is the mechanism regulating LMTK distribution, how do these mechanisms differ if at all in the case of different LMTKs? Is there crosstalk between them? Regarding to endosomal transport it is also a pressing question how LMTKs regulate it. For example, does LMTK1 directly phosphorylate TBC1D9B? Does LMTK2 recruit myosin VI to endosomes or other way around? Does LMTK2 phosphorylate myosin VI to regulate intracellular transport? Which cargoes are transported on and what targets them into LMTK-positive endosomes? Finally, LMTK1 and LMTK2 protein expressions are decreased in Alzheimer’s disease. Their loss can induce damages to axonal transport, affect synaptic signalling and promote amyloidogenic processing of APP and tau hyperphosphorylation which can lead to further transport and synaptic defects. Additionally, in the case of at least in LMTK2, reduced LMTK2 expression is an early pathological phenomenon in Alzheimer’s disease and early anomalies are generally believed to contribute the most to the disease pathogenesis. Although this review focuses on the link between LMTKs and neurodegenerative diseases it worth noting that recent studies also found potential associations between LMTKs and neurodevelopmental disorders, and there is now growing amount of evidence that damages to axonal transport and endosomal trafficking can contribute to the pathogenesis of these diseases as well [195, 196]. Thus, whole-exome sequencing found *LMTK1* as a candidate causative gene in global developmental delay/intellectual disability while *LMTK1* copy number gain has been identified in some schizophrenia patients [197, 198]. Furthermore, exome sequencing identified a de novo, potentially gene disrupting, *LMTK3* mutation in patients suffering in autistic spectrum disorders [199, 200]. Finally, significant *LMTK2* gene expression reductions have been observed in autism spectrum disorder and schizophrenia risk models [201, 202]. Thus, compromised LMTK expression and its affect on axonal transport and synaptic function might be involved in neurodevelopmental disorders as well which worth further investigation. Lots of questions raised but not enough answers have been found yet. However, one important direction must be highlighted and it is the challenge to explore whether LMTKs could be used as therapeutic targets to restore synaptic functions in neurodegenerative diseases.

## Data Availability

Not applicable.

## Abbreviations

ALS/FTD: Amyotrophic lateral sclerosis/frontotemporal dementia
AMPA: α-amino-3-hydroxy-5-methyl-4-isoxazolepropionic acid
APP: Amyloid precursor protein
BACE1: Beta-site APP cleaving enzyme-1
cdk5: Cyclin dependent kinase-5
CFTR: Cystic fibrosis transmembrane conductance regulator
GABA: Gamma-aminobutyric acid
GluA1: Glutamate ionotropic receptor AMPA-type subunit-1
GluN1 and GluN2B: Glutamate ionotropic receptor NMDA-type subunit-1 and 2B
GSK3β: Glycogen synthase kinase-3β
KCC2: Potassium-chloride cotransporter 2
KLC: Kinesin-1 light chain
LMTK: Lemur tail kinase
LTD: Long-term depression
LTP: Long-term potentiation
miRNA: Micro RNA
NF-κB: Nuclear factor-kappa B
NMDA: N-methyl-D-aspartic acid
Nrf2: Nuclear factor erythroid 2-related factor 2
NUSAP1: Nucleolar and spindle associated protein 1
PP1: Protein phosphatase-1
PP1C: Catalytic subunit of PP1
SH3: Sarcoma homology-3
siRNA: Small interfering RNA
Smad2: Mothers against decapentaplegic 2
Src: Sarcoma
TBC1D9B: Transferrin receptor-like 2-budding uninhibited by benzimidazoles-2-cell division cycle protein-16 homolog-1 domain family member-9B
TGF-β1: Transforming growth factor-β1
WE/WD motif: Tryptophan-glutamic acid/tryptophan-aspartic acid motif
